# Effects of soluble Klotho and Wnt/β-catenin signaling pathway in vascular calcification in chronic kidney disease model rats and the intervention of Shenyuan granules

**DOI:** 10.1080/0886022X.2024.2394633

**Published:** 2024-09-04

**Authors:** Xinrong Zou, Changjiang Wang, Lan Wang, Shenghua Huang, Danfang Deng, Lamei Lin, Xiaoqin Wang

**Affiliations:** a Department of Nephrology, Hubei Provincial Hospital of Traditional Chinese Medicine, Wuhan, China; b Hubei Key Laboratory of Theory and Application Research of Liver and Kidney in Traditional Chinese Medicine, Affiliated Hospital of Hubei University of Traditional Chinese Medicine, Wuhan, China; c Hubei Shizhen Laboratory, Wuhan, China; d Hubei Institute of Traditional Chinese Medicine, Wuhan, China

**Keywords:** Chronic kidney disease (CKD), vascular calcification, sKlotho, Wnt/β-catenin, Shenyuan granules

## Abstract

**Objective:**

This study aimed to investigate the effect of the soluble Klotho (sKlotho)/Wnt/β-catenin signaling pathway on vascular calcification in rat models of chronic kidney disease (CKD) and the intervention effect of Shenyuan granules.

**Methods:**

Rats with 5/6 nephrectomy and high phosphorus feeding were used to establish the vascular calcification model. The rats were given gradient doses of Shenyuan granules aqueous solution and calcitriol solution by gavage for 8 weeks, which were divided into experimental group and positive control group.

**Results:**

The 5/6 nephrectomy combined with high phosphorus feeding induced thoracic aortic calcification in rats. Shenyuan granules intervention increased the serum sKlotho level, inhibited the mRNA and protein expression of Wnt1, β-catenin, and Runx2 in the thoracic aorta, and alleviated thoracic aortic media calcification in rats.

**Conclusion:**

Shenyuan granules may partially regulate the Wnt/β-catenin signaling pathway via serum sKl to interfere with the expression of Runx2, thereby improving vascular calcification in CKD.

## 1. Introduction

The incidence of immune-mediated kidney diseases remains high, and the number of different types of kidney disease caused by metabolic diseases, such as diabetes, is increasing daily, leading to a rapid rise in the incidence of chronic kidney disease (CKD) worldwide [1]. According to recent studies, the global prevalence of CKD is about 9.2% [2]. The reported prevalence of CKD in China in 2018 was 11.6% [3], it has become a serious public health concern.

Cardiovascular disease (CVD), a serious complication in CKD, is the main cause of death in patients with CKD [4]. The results of a multicenter prospective cohort study on Chinese patients with CKD (C-STRID) showed that the baseline prevalence of aorta abdominalis calcification in patients with stage 1–4 CKD was 9.22%, and aorta abdominalis calcification was one of the independent risk factors for CVDs in patients with CKD [5]. Vascular calcification increases in severity as the estimated glomerular filtration rate decreases. The Chinese Dialysis Calcification Study revealed that the prevalence of vascular/cardiac valve calcification in patients undergoing hemodialysis and peritoneal dialysis is as high as 77.4%, thereby greatly increasing the risk of CVD [6]. Vascular calcification in CKD is a complex pathological progression process in which many signal factors participate jointly in its regulation [7]. Studies have confirmed that the decrease in the level of Klotho (Kl) is an important reason for vascular calcification in CKD [8], and serum soluble Kl (sKl) is an endocrine factor that can play a variety of biological effects when membrane form Kl is sheared and released into the blood [9].

The Wingless/Integrated (Wnt) signaling pathway activation participates in vascular calcification by promoting the expression of osteogenic differentiation-specific transcription factor (Runx2) in vascular smooth muscle cells (VSMCs) [10]. Although calcitriol is widely used as a common regulator of calcium (Ca), phosphorus (P), and parathyroid hormone (PTH) in clinical practice, there is still a lack of effective prevention and treatment methods for vascular calcification.

The application of traditional Chinese medicine (TCM) in improving CKD have gained enormous attentions of scholars and some TCM have been demonstrated to play an important role in delaying the progress of CKD by regulating multiple targets and pathways. Shenyuan granules, a kind of TCM formula, possessed the effects on tonifying the spleen and kidney, removing blood stasis and clearing turbidity on base of TCM theory. Previous study have showed that Shenyuan granules have potential of improved Ca and P metabolism and renal function in remnant kidney model rats by upregulating the expression of kidney Kl [11], and alleviated kidney damage in diabetic nephropathy mice by inhibiting the Wnt signaling pathway [12]. In this study, we aimed to further investigate the effects and mechanisms of action of the changes in serum sKl levels on Wnt/β-catenin expression and vascular calcification in remnant kidney rats and identify the possible targets of Shenyuan granules in the intervention of vascular calcification in CKD.

## 2. Materials

### 2.1. Laboratory animals

The laboratory animals were 6-week-old male specific-pathogen-free Sprague–Dawley (SD) rats, with a body mass of 170 ∼ 200 g. They were purchased from Beijing Vital River Laboratory Animal Research Center (License: SCXK [Beijing] 2016-0006; Certificate No. 110011201109380827). The rats were raised in a barrier environment at a temperature of 20–24 °C and humidity of (40 ± 10%). They were given free access to water and food.

### 2.2. Main drugs and reagents

The following were used: Shenyuan granules (manufactured from *Astragali radix*, *Herba epimedii*, and *Rheum officinale* at the preparation center of Hubei Provincial TCM Hospital [Approval No. EYZZ Z20180053]); calcitriol soft capsules (CP Pharmaceutical [Qingdao] Co., Ltd. [GYZZ H20030491]); RNA extract, RNA reverse-transcription (RT) kit, and all-in-one quantitative polymerase chain reaction (qPCR) mix (Wuhan Servicebio Technology Co., Ltd.); rabbit anti-Wnt1 (ABclonal, USA); rabbit anti-β-catenin and mouse anti-actin (Wuhan Servicebio Technology Co., Ltd.); rabbit anti-Runx2 (Beijing BIOSS); rabbit anti-Sm22α (Wuhan Sanying Biology Technology Co., Ltd.); rabbit anti-osteocalcin (OCN) (Abcam, UK); horseradish peroxidase (HRP)-conjugated secondary antibody (Wuhan Servicebio Technology Co., Ltd.); a Roche Biochemical Kit (Roche, Switzerland); a chemiluminescence immunoassay kit (Siemens, USA), and an sKl ELISA kit (Shanghai Enzyme-linked Biotechnology Co., Ltd.).

## 3. Methods

### 3.1. Modeling, grouping, and administration

The SD rat models were established after adaptive feeding for 7 days. The remnant kidney rat model was established by 5/6 nephrectomy, as follows: After anesthesia by intraperitoneal injection of 3% pentobarbital sodium (3 mg/100 g), the skin beside the spine was trimmed, sterilized, and cut diagonally downward about 2 cm at a distance of 1 cm below the left costalspinal angle on the back. The subcutaneous tissues and fascias were separated layer by layer. The left kidney was exposed, the renal capsule was peeled off, and the upper and lower poles of the left kidney were ligated and resected. After no bleeding was confirmed, the incision was sutured. High-P drinking water (1.2% sodium dihydrogen phosphate solution) was administered on the second day after surgery. One week later, an incision was made under the right costalspinal angle to separate and ligate the right renal artery and the right ureter; then, the right kidney was removed, and the incision was sutured.

After successful model establishment, rats were fed with high-P water until the termination of experiments. the rats were divided into a nephrectomized control group (NC, *n* = 10), positive control group (PC, *n* = 10), low-dose Shenyuan group (SYG-L group, *n* = 10), and high-dose Shenyuan group (SYG-H group, *n* = 10) using a random number table. A normal group (normal, *n* = 10), which was provided separately, was given water normally. Eight weeks after modeling surgery, gastric perfusion was started for each group of rats. The drug dosage of the rats was converted by the body surface coefficient. The reduction coefficient of human and rat body surface area of 0.018, and 60 kg adults were given 30 g of Shenyuan granules orally daily [13]. The normal group and the NC were given distilled water for gastric perfusion, the PC was given calcitriol suspension for gastric perfusion (including 0.003-µg/ml calcitriol), the SYG-L group was given 0.35-g/ml aqueous solution of Shenyuan granules for gastric perfusion, and the SYG-H group was given 0.7-g/ml aqueous solution of Shenyuan granules for gastric perfusion, Only 1 ml/100 g/day of Shenyuan granules was used in gastric perfusion, once a d, and gastric perfusion was continued for 8 weeks.

### 3.2. Collection and processing of specimens

Before administration, blood was taken from the orbit for the detection of blood urea nitrogen (BUN) and serum creatinine (SCr). After gastric perfusion, blood was collected from the orbits of the rats in each group and centrifuged, with the serum retained for the detection of various biochemical and serum indexes. At the end of the experiment, the rats were euthanized by decapitation. Their thoracic cavities were opened to separate the thoracic aortas, which were cut into segments of about 0.5 cm in length. Some of aortas or the segments were put into a −80 °C refrigerator for cryopreservation for RT-qPCR, Western blot, and immunohistochemical detection, and some were placed into a 4% paraformaldehyde solution for dedicated Von Kossa staining.

### 3.3. Test indexes

#### 3.3.1. Detection of biochemical indexes

A Roche automatic biochemical analyzer was used for the detection of BUN, SCr, serum Ca, P, and alkaline phosphatase (ALP), and a Siemens chemiluminescence immunoanalyzer was used for the detection of serum intact parathyroid hormone (iPTH).

#### 3.3.2. Detection of sKl by ELISA

Standard and sample holes were provided, respectively, and 50-µl standard samples of different concentrations were added to the standard holes along with 50 µl of the sample being tested. Then, 100 µl of HRP-labeled antibody was added to each of the standard and sample holes. The reaction hole was sealed with sealing film and incubated at 37 °C for 60 min. The liquid was discarded, the hole was filled, and it was dried with absorbent paper. Each hole was filled with 350 µl of washing liquid and left to stand for 1 min. Then, the washing liquid was discarded, and the hole was dried with absorbent paper. The washing process was repeated five times. Next, 50 µl of substrate A and 50 µl of substrate B were added to each hole and incubated at 37 °C in the dark for 15 min. Finally, 50 µl of stop solution was added to each hole, and the optical density (OD) value of each hole was measured at a wavelength of 450 nm within 15 min.

#### 3.3.3. Observation of thoracic aorta calcification by Von Kossa staining

The thoracic aorta tissue fixed by paraformaldehyde was removed for gradient alcohol sequential dehydration. After xylene transparency, paraffin embedding, sectioning, and drying were performed. A solution of 1% silver nitrate was added dropwise into the section for dip dyeing. Then, it was irradiated under an ultraviolet lamp for 2–3 h to remove the silver nitrate solution, after which, 5% sodium thiosulfate aqueous solution was added and left for 1 min. Counterstaining was performed with 0.5% neutral eosin solution for 10 s; then, the samples were dehydrated and mounted on neutral resin. Calcification staining was observed under a microscope, with black granules presented in the tunica media of the thoracic aorta.

#### 3.3.4. Detection of mRNA of Wnt1, β-catenin, Runx2, Sm22α, and OCN in thoracic aortas by RT-qPCR

A 100-mg volume of thoracic aortic tissue was added to the precooled 1-ml RNA extraction solution, and the total RNA of each group of thoracic aortas was extracted according to the manufacturer’s instructions. The concentration and purity of the RNAs were detected using Nanodrop 2000. The RNAs were diluted with an excessive concentration in an appropriate proportion to establish a final concentration of between 100 and 500 ng/µl. RT was conducted to prepare the RT system (20 µl). The reaction conditions were 25 °C for 5 min, 42 °C for 30 min, and 85 °C for 5 min. Next, 2 × 7.5-µl qPCR mix, 2.5-μM gene primer (1.5 µl), 2.0-µl RT (cDNA), and ddH_2_O (4.0 µl) were added to a 0.2-ml PCR tube. Three tubes were prepared for each cDNA.

For the PCR amplification, the reaction conditions were 90 °C for 10 min, 95 °C for 15 min, and 60 °C for 30 s. Forty cycles were performed at 65–95 °C, with a fluorescence signal collected every 0.5 °C/s to plot the dissolution curve. The final data were analyzed using 2^−△△Ct^. The primer sequence was designed using Primer 5.0 software (Table 1).

**Table 1.** Information of main primer of RT-qPCR.

Primer name		Primer sequence (5′–3′)	Segment length (bp)
Wnt1	F	GGGACCTACGCTTCCTCATG	96
	R	ACATCCCGTGGCATTTGCA	
β-catenin	F	TGCCATCTGTGCTCTTCGTC	151
	R	CAATCCAACAGTTGCCTTTATCAG	
Runx2	F	CAGTATGAGAGTAGGTGTCCCGC	152
	R	AAGAGGGGTAAGACTGGTCATAGG	
Sm22α	F	GTAATGGCTTTGGGCAGTTTG	109
	R	TGTCTGTGAACTCCCTCTTATGCT	
OCN	F	TGACAAAGCCTTCATGTCCAA	80
	R	CTCCAAGTCCATTGTTGAGGTAG	
GAPDH	F	CTGGAGAAACCTGCCAAGTATG	138
	R	GGTGGAAGAATGGGAGTTGCT	

#### 3.3.5. Protein expression of Wnt1, β-catenin, Runx2, Sm22α, and OCN in detection of thoracic aortas by Western blot

About 100 mg of thoracic aorta tissue was added to 10 times its volume of protein lysate for homogenization; it was fully lysed in a homogenizer and centrifuged at 4 °C for 10 min at 12,000 rpm. The supernatant was collected as the total protein solution. The protein solution was added to a reduced protein-loading buffer at a ratio of 4:1. Then, it was denatured in a bath of boiling water for 15 min and placed in a refrigerator at −20 °C for later use.

To prepare the electrophoresis gel, 50 µg of the total protein sample was extracted, and electrophoresis was conducted for about 1.5 h. The gel was removed, the target strip was cut according to the label, and the electrotransfer buffer was filled. A polyvinylidene fluoride (PVDF) membrane was transferred to the membrane transfer tank. Then, the transferred PVDF membrane was placed into an incubation tank with Tris buffered saline with Tween-20 (TBST) for washing. Next, it was put in a shaker containing 5% skimmed milk powder for blocking at room temperature for 30 min. The blocking buffer was discarded, and the prepared primary antibody was added and incubated overnight at 4 °C. The membrane was washed with TBST three times, and the HRP-labeled secondary antibody was diluted with TBST at a ratio of 1:5000. It was incubated at room temperature for 30 min, and the membrane was washed a further three times. The mixed enhanced chemiluminescence solution was transferred to the PVDF membrane for gel pressing, development, exposure, and film scanning, and the OD value of the target strip was analyzed using the Alpha software^™^ processing system.

#### 3.3.6. Distribution of Wnt1, β-catenin, Runx2, Sm22α, and OCN in immunohistochemical detection of thoracic aortas

The thoracic aorta tissue was subjected to dehydration, transparency, waxing, embedding, paraffin sectioning, baking, and dewaxing. Prepared 3% hydrogen peroxide was dropped onto the sections and incubated at room temperature for 15 min. Then, it was washed with phosphate buffer saline (PBS) three times. The primary antibody was added at 4 °C overnight. Then, it was washed with PBS a further three times. Polymerization promoter was added and incubated at room temperature for 20 min before washing with PBS three times. HRP-labeled secondary antibody was added and incubated for 45 min. Then, it was washed with PBS, and a freshly prepared diaminobenzidine coloring solution was added. After coloring, Harris hematoxylin counterstaining, dehydration, and mounting were performed. The samples were observed under a microscope, and photographs were taken. Brown or light brown indicated positive expression.

## 4. Statistical methods

The data were processed with the SPSS 19.0 statistical software package. The measurement data were expressed in ($\bar{X}$ ± S). An analysis of variance (ANOVA) was used for comparisons between groups, an ANOVA test and Levene’s test for homogeneity of variances were used for mean comparisons, and the least-significant difference method and the Tamhane’s T2 method were used for pairwise comparisons. A value of *p* < 0.05 indicated a difference with statistical significance.

## 5. Results

### 5.1. Detection results of blood biochemical indexes, iPTH, and serum sKl

After 8 weeks of administration, the blood BUN, SCr, iPTH, and Ca levels of the NC and the PC were significantly higher than those of the normal group. The blood BUN, SCr, iPTH, and Ca of the SYG-L group and the SYG-H group were lower than those of the NC, and the blood BUN, SCr, and Ca of the SYG-L group and the SYG-H group were also lower than those of the PC, representing a statistically significant difference. The serum P levels in the NC and the PC were higher than that of the normal group, while those of the SYG-L group and the SYG-H group were lower than those of the PC; however, there was no significant difference compared with the NC. There was no significant difference in blood ALP among the groups.

According to the ELISA results, the levels of serum sKl in the nephrectomized group and the PC were significantly lower than those in the normal group. The levels of sKl in the SYG-L group and the SYG-H group were significantly higher than those in the nephrectomized group, and the level of sKl in the SYG-H group was higher than that in the PC, representing a statistically significant difference (Table 2).

**Table 2.** Test results of blood BUN, SCr, Ca, P, ALP, PTH, and sKl of rats in each group.

Group	*n*	Bun (mmol/L)	SCr (μmol/L)	Ca (mmol/L)	P (mmol/L)	ALP (U/L)	iPTH (pg/ml)	sKl (pg/ml)
Normal group	10	5.0 ± 0.8	26.2 ± 8.4	2.2 ± 0.2	1.5 ± 0.3	95.5 ± 24.9	1.1 ± 0.2	16.4 ± 2.7
NC	10	18.3 ± 4.3^a^	92.5 ± 28.9^a^	2.6 ± 0.1^a^	1.8 ± 0.4^a^	123.0 ± 45.1	1.5 ± 0.2^a^	5.8 ± 3.7^a^
PC	10	20.9 ± 9.5^a^	91.4 ± 45.6^a^	2.7 ± 0.2^a^	2.3 ± 0.3^a^	128.4 ± 58.6	1.5 ± 0.2^a^	9.5 ± 2.0^a^
SYG-L group	10	9.4 ± 1.8^b^^,^^c^	50.3 ± 7.8^b^^,^^c^	2.1 ± 0.2^b^^,^^c^	1.6 ± 0.2^c^	101.2 ± 37.7	1.2 ± 0.2^b^^,^^c^	11.0 ± 1.9^b^
SYG-H group	10	11.5 ± 3.3^a–c^	50.0 ± 17.4^b^^,^^c^	2.0 ± 0.2^b^^,^^c^	1.6 ± 0.4^c^	84.2 ± 21.7	1.0 ± 0.1^b^^,^^c^	15.4 ± 4.3^b^^,^^c^

^a^ Compared with the normal group, *p* < 0.05.

^b^ Compared with the NC, *p* < 0.05.

^c^ Compared with the PC, *p* < 0.05.

### 5.2. Calcification of thoracic aortas of rats in each group after Von Kossa staining

**Figure 1.**
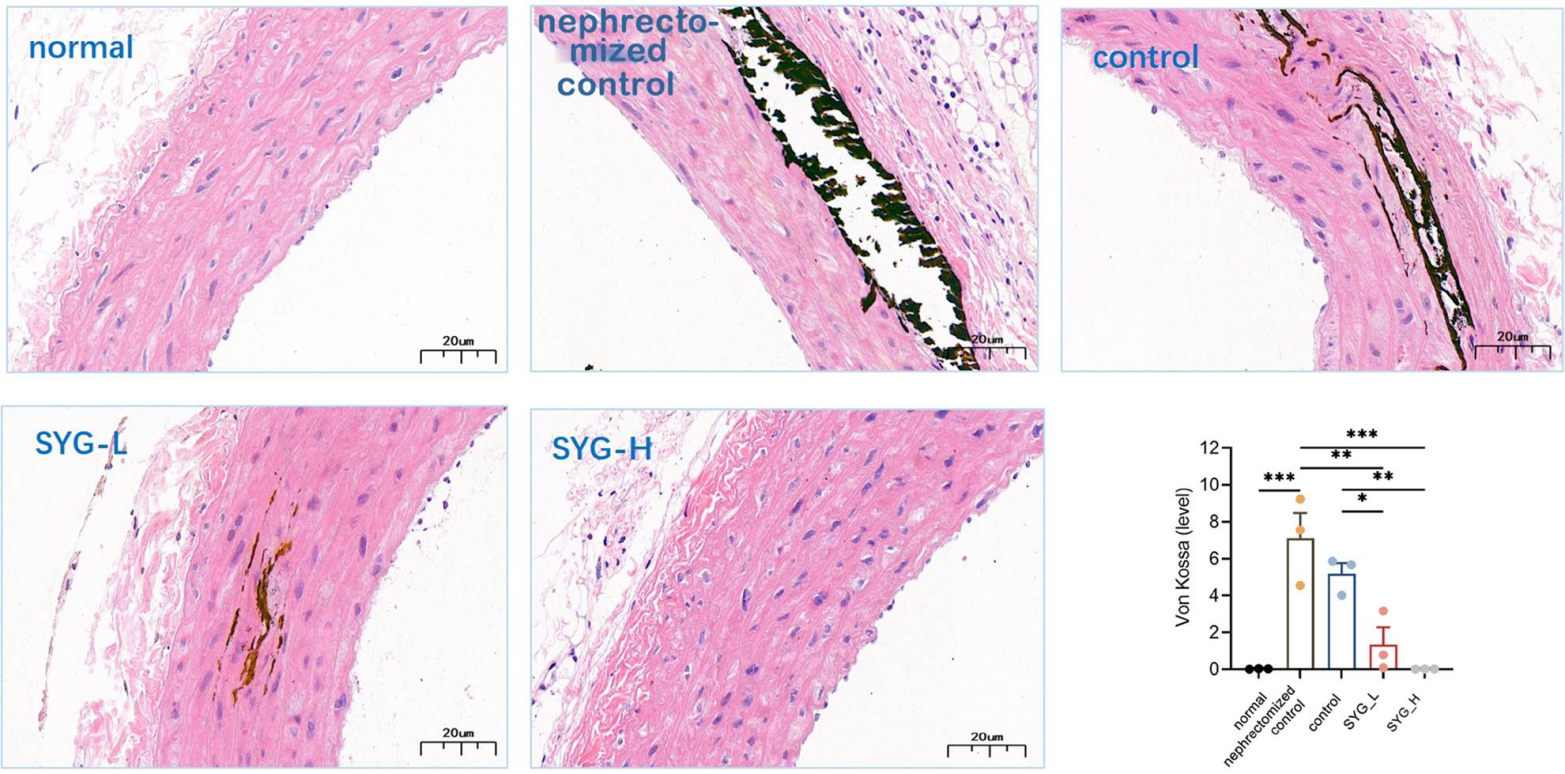
Calcification and quantitative assessment of thoracic aortas of rats in each group after Von Kossa staining (400×).

Under the optical microscope, it was found that the thoracic aortas of the rats in the normal group had tight vascular walls and smooth intimae, and the VSMCs in the tunica media were arranged regularly, without Ca deposition. In the nephrectomized group, intimal hyperplasia was present in the vessel walls, the structure of each layer was loose, the VSMCs were arranged irregularly, and there was a large amount of Ca deposition stacked in the tunica media. The vascular changes in the PC were similar to those in the nephrectomized group. Compared with the nephrectomized group and the PC, the vascular intimal hyperplasia in the SYG-L group was alleviated, the arrangement of VSMCs was improved, and punctate and linear Ca deposition was scattered in the tunica media. The structure of each layer in the vascular walls of the SYG-H group was significantly improved compared with that of both the nephrectomized group and the PC, with only a little punctate Ca deposition scattered in the tunica media (Figure 1). Meanwhile, the quantitative assessment of Von Kossa staining is visualized in Figure 1.

### 5.3. mRNA expression of Wnt1, β-catenin, Runx2, Sm22α, and OCN in RT-qPCR detection

**Figure 2.**
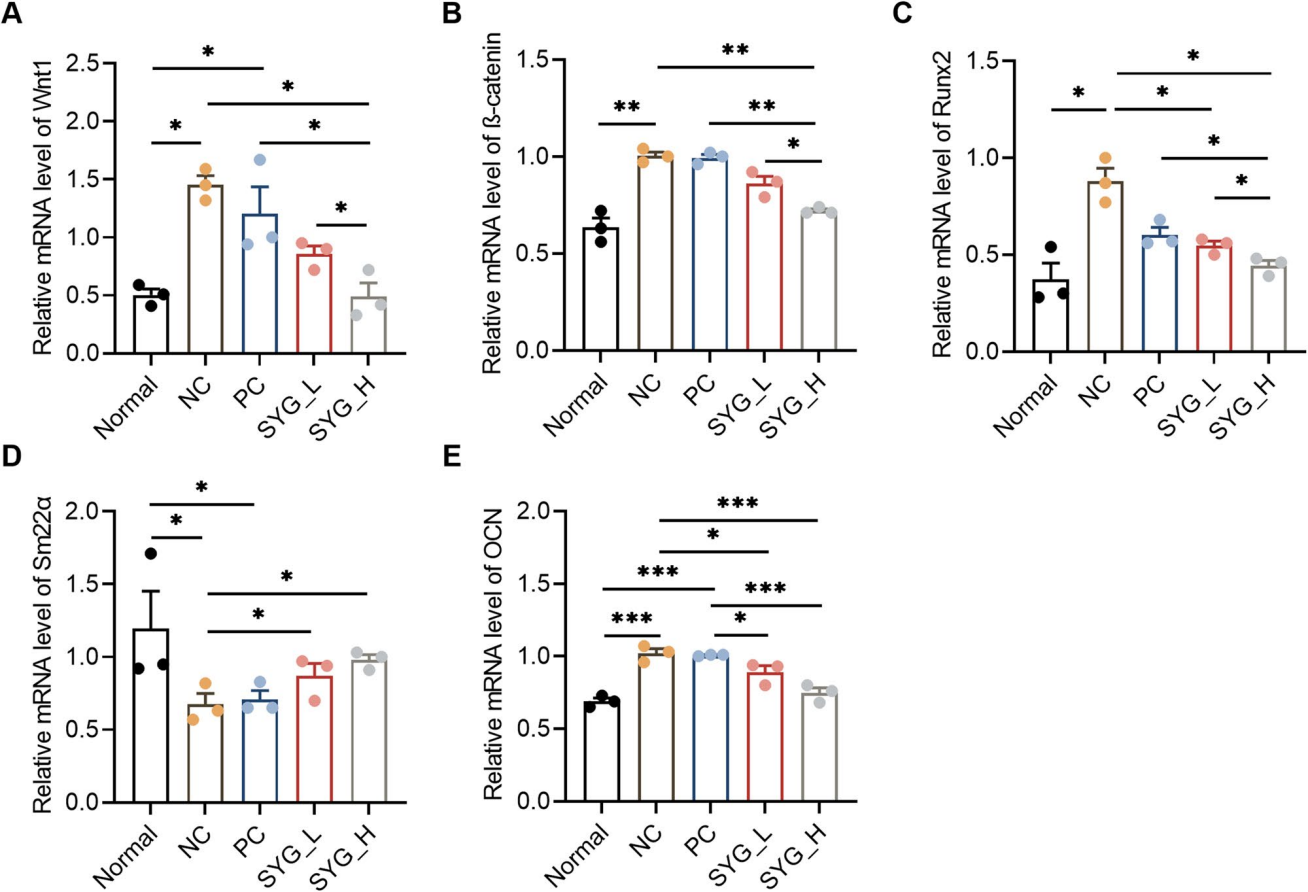
mRNA Expression of Wnt1, β-catenin, Runx2, Sm22α, and OCN in thoracic aorta were detected by RT-qPCR (A–E) (*n* = 3). *p﹤0.05, **p﹤0.01, ***p﹤0.001.

Compared with the normal group, the mRNA expression of Wnt1, β-catenin, Runx2, and OCN in the thoracic aortas of the nephrectomized group was enhanced, while that of Sm22α was decreased. The mRNA expression of Wnt1 and OCN was enhanced in the PC, while that of Sm22α was decreased, with a statistically significant difference (Figure 2).

Compared with NC group lower expression of Wnt1, β-catenin, Runx2, and OCN, as well as higher expression of Sm22α were observed in the high-dose nephron group. Lower expression of Runx2 and OCN, as well as higher expression of Sm22α were observed in the low-dose nephron group. Only OCN expression was decreased compared with the PC.

### 5.4. Protein expression of Wnt1, β-catenin, Runx2, Sm22α, and OCN in detection of thoracic aortas by Western blot

**Figure 3.**
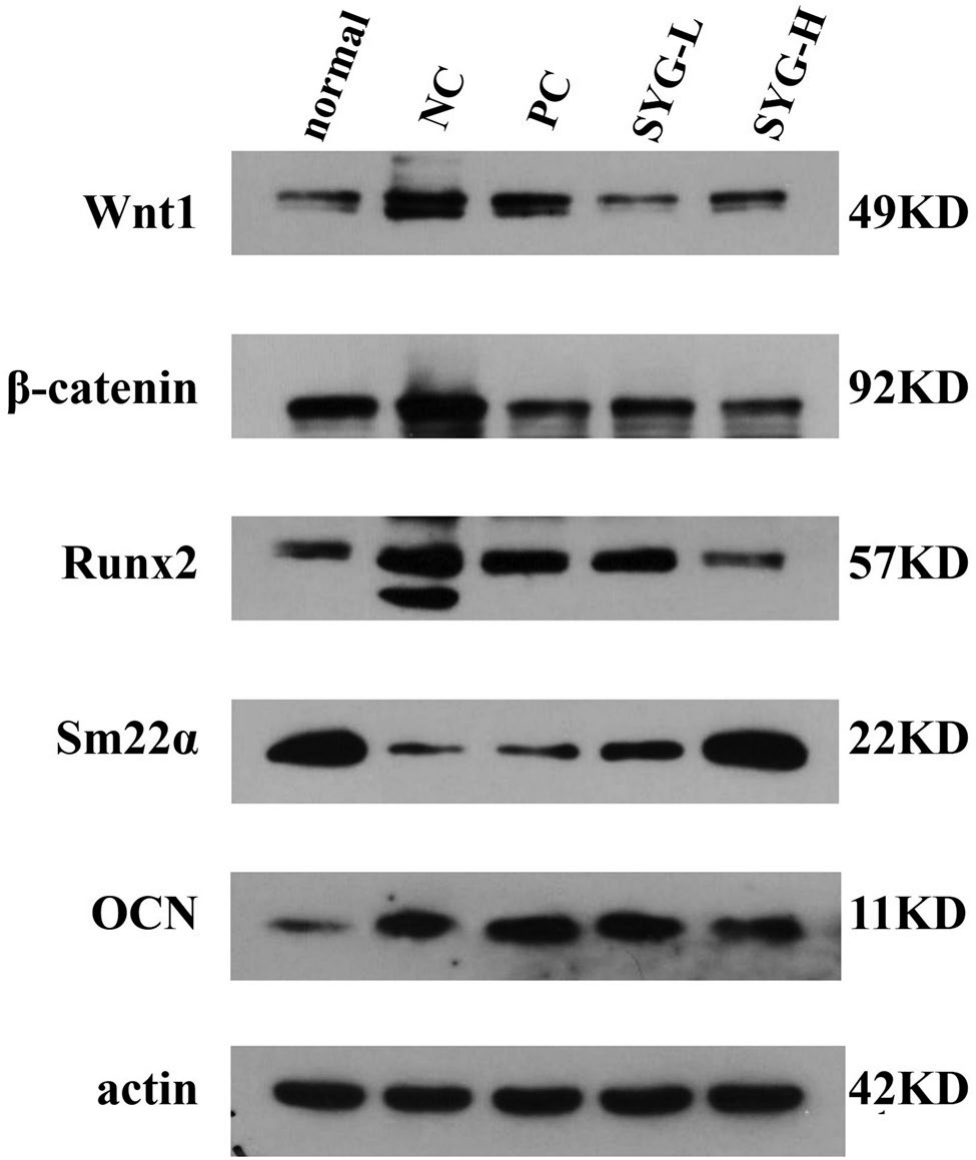
Protein expression of Wnt1, β-catenin, Runx2, Sm22α, and OCN in Western blot detection of thoracic aortas of rats in each group.

**Figure 4.**
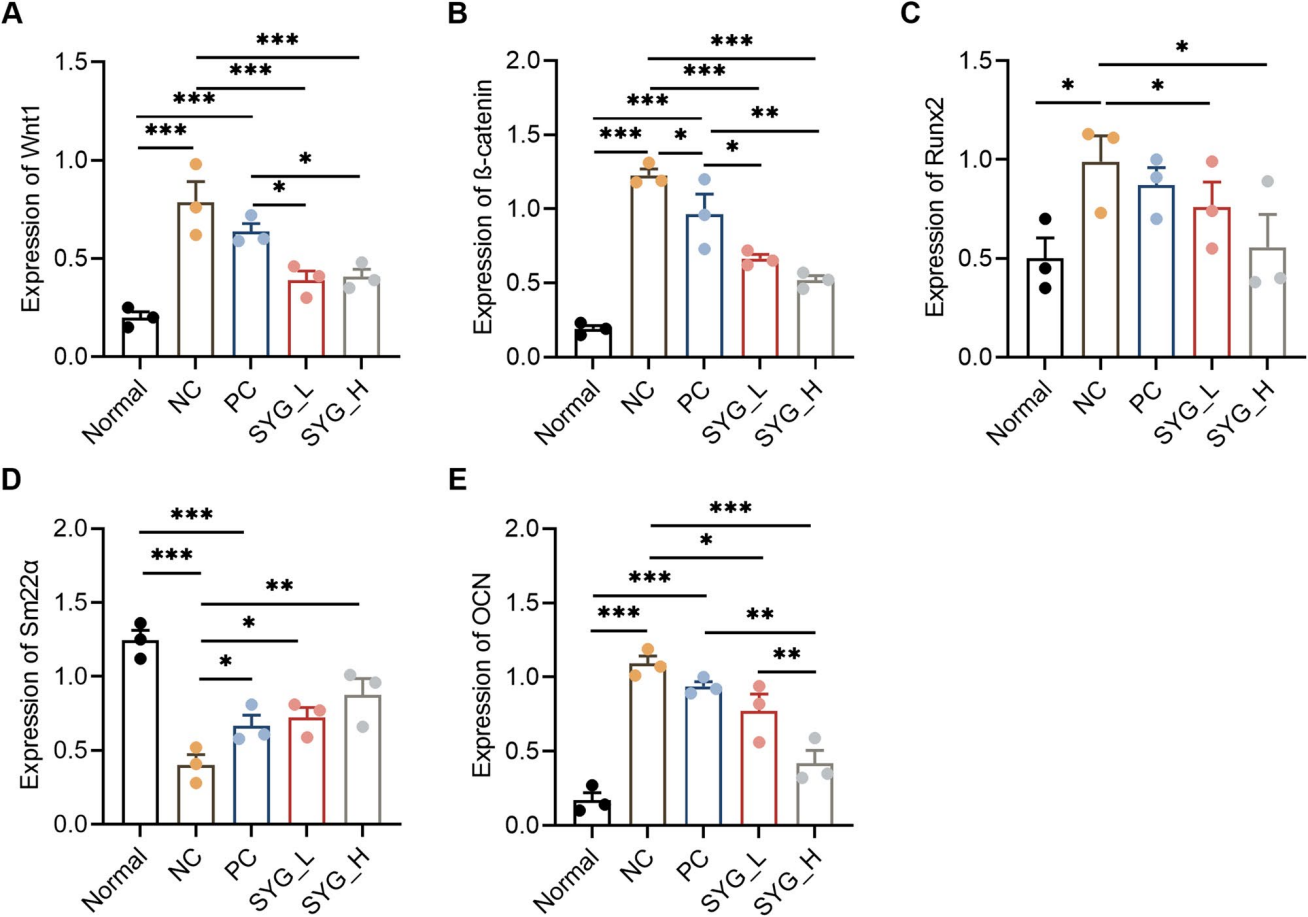
Relative amount of protein expression of Wnt1, β-catenin, Runx2, Sm22α, and OCN in Western blot detection of thoracic aortas of rats in each group (A–E) (*n* = 3). *p﹤0.05, **p﹤0.01, ***p﹤0.001.

The expression of Wnt1, β-catenin, Runx2, and OCN in the thoracic aortas of the nephrectomized group was increased compared with that of the normal group, while the expression of Sm22α was reduced. The expression of Wnt1, Runx2, and OCN in the PC was increased, while the expression of Sm22α was decreased, with a statistically significant difference. The expression of Wnt1, β-catenin, Runx2, and OCN in the SYG-L group and the SYG-H group was decreased compared with that of the model group, but the expression of Sm22α was increased. Compared with the PC, the expression of Wnt1, β-catenin, and OCN in the SYG-L group and the SYG-H group was decreased, but there was no statistical difference among other the indexes. Compared with the SYG-L group, the expression of OCN in the SYG-H group was decreased, and there was no statistical difference among the other indexes (Figures 3–4).

### 5.5. Distribution of Wnt1, β-catenin, Runx2, Sm22α, and OCN in immunohistochemical detection of thoracic aortas of the rats in each group

**Figure 5.**
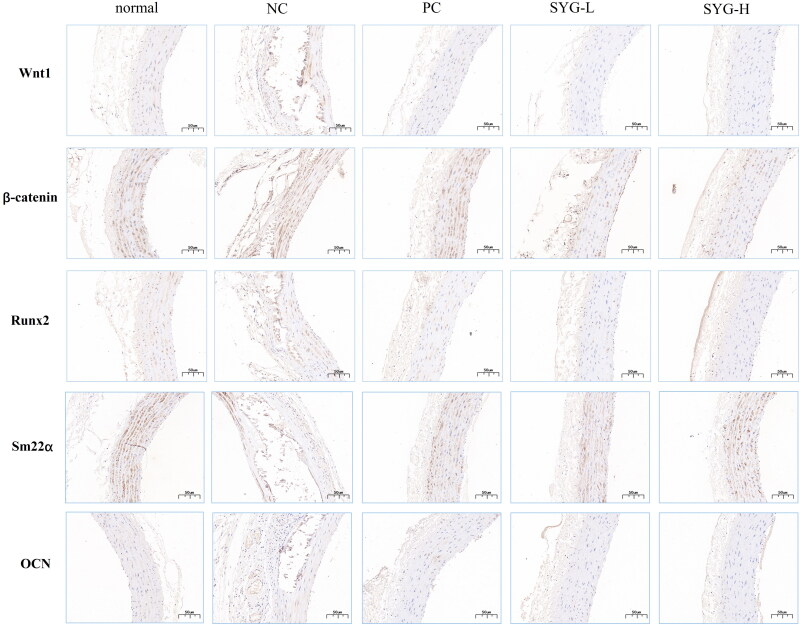
Immunohistochemical staining results of Wnt1, β-catenin, Runx2, Sm22α, and OCN in thoracic aortas of rats in each group (200×).

The immunohistochemical results of the rats in each group revealed that the staining intensity of Wnt1, β-catenin, Runx2, and OCN in the thoracic aortas of the nephrectomized group and the PC was higher than that of the normal group, while the staining intensity of Sm22α was lower. The staining intensity of Wnt1, β-catenin, Runx2, and OCN in the SYG-L group and the SYG-H group was decreased compared with that in the model group, while the Sm22α staining intensity was increased, particularly in the SYG-H group (Figure 5).

## 6. Discussion

As a complex pathological process similar to bone development [7], vascular calcification indicates a higher cumulative incidence of myocardial infarction and heart failure, which is positively correlated with cardiovascular and all-cause mortality risks [14]. Vascular calcification may occur in the intima and tunica media of blood vessels, with tunica media calcification as the main manifestation of vascular calcification in patients with CKD [15]. The main functional units of arterial tunica media are VSMCs. The transformation of VSMCs from a contractile phenotype to an osteoblastic phenotype is an important feature of vascular calcification in CKD. Under the regulation of upstream factors, the expression of Runx2 in VSMCs is enhanced, bone matrix-like components, such as OCN and osteopontin, are increased, muscle cell markers, such as alpha smooth muscle actin (Sm22) are decreased [16,17], and Ca disposition occurs in cells and tissues. Hypercalcemia, hyperphosphatemia, and a high concentration of PTH associated with CKD are the key factors that induce phenotypic changes to form tunica media calcification [18,19].

Associated with anti-aging, the *Kl* gene is expressed mainly in the kidney, parathyroid gland, and the choroid of the brain. Deficiency of the *Kl* gene is manifested as atherosclerosis and progeria [20]. The Kl proteins encoded by the *Kl* gene can be divided into membrane-bound and secretory types. sKl, which is sheared and released into the bodily fluids by the extracellular domain of membrane type Kl, exists mainly in the cerebrospinal fluid, blood, and urine of animals. sKl is a pleiotropic endocrine factor [8,9]. The decrease in sKl levels is an independent predictor of renal disease progression [21,22]. Studies on vascular calcification in patients on maintenance hemodialysis have shown the sKl level to be significantly negatively correlated with the incidence of aorta abdominalis calcification [23]. In studies on animal models, it was found that the Wnt signaling pathway was involved in vascular calcification and the osteogenic transformation of VSMCs [24,25], and the Wnt/β-catenin pathway plays an important role in hyperphosphate-induced VSMC calcification [26]. In the skin, blood vessels, bones, and other tissues and organs of Kl-deficient mammals, Wnt signal expression is significantly enhanced. Continuous Wnt exposure accelerates the senescence of tissues and cells, while the ectopic expression of Kl can inhibit endogenous and exogenous Wnt activity [27]. Another study found increased expression of β-catenin in aorta of Klotho mutant (kl/kl) mice. The coculture of Wnt3a and human aortic VSMCs was observed with the increased expression of Runx2, early osteogenic genes, and BMP4, which indicated the cell calcification. The process could be alleviated by the interference of eicosapentaenoic acid [10]. Therefore, in order to further explore intervention targets and drugs for vascular calcification, our study intends to observe whether changes in circulating sKl level can regulate the activity of Wnt/β-catenin signaling pathway in arterial vascular tissue and thus affect vascular calcification in the CKD model, as well as the intervention effect of TCM on vascular calcification.

In this study, rat models of vascular calcification in CKD were successfully constructed by performing 5/6 nephrectomy and administering high-P drinking water. In the model group, the serum sKl level was significantly reduced, the levels of Ca, P, and PTH in the blood were increased, the mRNA and protein expression of Wnt1, β-catenin, Runx2, and OCN in the thoracic aorta tissues was enhanced, the mRNA and protein expression of Sm22α was weakened, a large amount of Ca salt was deposited on the aortic tunica media, and calcification occurred in the arterial vessels. After intervention with low- and high-dose Shenyuan granules, the sKl level increased, the increased levels of blood Ca, P, and PTH were alleviated, the mRNA and protein expression of Wnt1, β-catenin, Runx2, and OCN in the thoracic aorta tissues decreased, the mRNA and protein expression of Sm22α increased, the Ca deposition in aortic tunica media decreased, and the calcification in arterial vessels was significantly reduced, especially after the high-dose administration of Shenyuan granules. However, there was no significant improvement in vascular calcification in the PC after the administration of calcitriol. Calcitriol contributed to the increase of sKl levels, and the higher sKl level in high-dose Shenyuan granules was detected. Compared with calcitriol, Shenyuan granules not only down-regulated the mRNA level of Wnt1, β-catenin, Runx2, and OCN, but also down-regulated the protein level of Wnt1, β-catenin, and OCN.

In TCM theory, spleen–kidney deficiency (partly caused by spleen and stomach digestive dysfunction) and vessel obstruction by turbidity and stasis (the harmful substances, such as blood stasis, phlegm, and dampness gathered together, resulting in blocking the meridians of body and finally causing disease) have been considered as the crucial influenced factors on the pathogenesis of vascular calcification in CKD [28]. *Huangdi Neijing*, the ancient Chinese medical book completed before third century AD in China, has recorded that ‘the kidney stores the essence of life’ and ‘the kidney dominates bones’. The kidney above-mentioned possessed different meaning between the perspective of TCM and western medicine. In detail, kidney means one of the five ‘zang’ in TCM theory, not just an organ in body. The above-mentioned recordings mean that in TCM theory, ‘kidney’ is the root of the congenital constitution (e.g., immunity), which seals and stores the vital essence. The essence of the kidney generates the kidney qi, which promotes the growth and development of the human body by boosting the physiological functions of the viscera. Some scholar suggested that the stem cells and its function was similar to ‘kidney essence’. For example, bone-marrow mesenchymal stem cells (BMSCs, ‘kidney essence’) plays important role in in maintaining bone metabolism (‘kidney’ dominates bones.). The BMSCs with normal function (filling ‘kidney essence’) could be induced to differentiate into osteoblasts, chondrocytes, adipocytes, etc., which further promote bone formation. Instead, the BMSCs with abnormal function (deficiency of kidney essence) will led to unpredictable influence in bone formation. Under the condition of CKD, in the early stage of CKD, there is a deficiency of kidney essence and kidney qi, which manifests as fatigue, edema, proteinuria, and hematuria. With the progression of the disease, the function of the ‘kidney dominating bone’ is weakened (the BMSCs showed abnormal function), which manifests as soreness and weakness of the waist and knees as well as weakness and pain in the bones. In the later stage of CKD, it develops into a series of changes, for example spleen and stomach digestive dysfunction, decreased physical function, increased disease-related factors such as inflammation factors, and obstruction of collaterals. The clinical manifestations include Ca and P metabolism disorder, increased PTH, and the occurrence of vascular calcification and CVD events, which are very similar to Kl-defective phenotypes, such as growth retardation, reproductive organ atrophy, osteoporosis, vascular sclerosis, and shortened life span [20,29].

Shenyuan granules (formerly known as Shen’an granules) are a hospital preparation established by our department through long-term clinical practice, which formed according to the TCM pathogenesis characteristics of CKD that above-mentioned. A series of basic and clinical studies were conducted in the early stage of establishing this drug [12,30,31]. Shenyuan granules was formulated based on the TCM theory of ‘Jun’ (emperor)–’Chen’ (minister)–’Zuo’ (assistant)–’Shi’ (courier). In Chinese formula, ‘Jun’ herb mainly play role in neutralizing the main symptoms of the disease. ‘Chen’ drug exerts effects on supporting ‘Jun’ herb and strengthening the defense mechanism triggered by the action of ‘Jun’ herb. ‘Zuo’ herb was used for neutralizing the side damages induced by ‘Jun’ or ‘Chen’ as well as assisting the ‘Jun’ drug for subordinate symptoms. ‘Shi’ drug performed effects on delivering the others’ drugs to the location of disease. In Shenyuan granules, *A. radix*, *H. epimedii* and liquor-processed *R. officinale* acts as the ‘Jun’ drug, ‘Chen’ drug, and ‘Shi’ drug, respectively. Previous studies have shown that icariin promote bone metabolism, protect vascular endothelium and improve the vasomotor function of diabetic mice [32]. Emodin has a critical role in anti-inflammation, anti-oxidation, lipid regulation, anti-apoptosis, and vascular protection [33]. Our previous studies on remnant kidney rat models showed that in the case of CKD, the expression of renal Kl was reduced, the metabolism of Ca, P, and bone was disturbed, and Shenyuan granules improved Ca and P metabolism and renal function by upregulating the expression of Kl in the kidney [11]. Previous study found that diabetic nephropathy mice has the down-regulated Klotho and up-regulated Wnt1 and β-catenin in protein and mRNA level, exasperate damage containing proteinuria, lipid disorders, and pathological hurt of kidney tissue. After intervention of Shenyuan granules, Kl expression of kidney tissue was up-regulated, and the activities of Wnt1 and β-catenin were decreased, followed by proteinuria and pathological damage of kidney tissue was reduced [3]. Db/db mice was used to feed with high P to make diabetic vascular calcification model and the enhanced renal Kl expression and reduced vascular calcification were observed after Shenyuan granule intervention [12].

In the present study, the intervention effect of Shenyuan granules on serum sKl and vascular calcification was confirmed by constructing models of vascular calcification in CKD. The research results showed that with a decrease in the level of sKl, the activated Wnt/β-catenin signal in arterial tissue, overexpression of Runx2 and OCN, downexpression of Sm22α, and the occurrence of tunica media were observed. It was speculated that Shenyuan granules might reduce vascular calcification in CKD via partially upregulating the sKl level and inhibiting the sKl/Wnt/β-catenin pathway.

The high risk of CVDs caused by vascular calcification in CKD cannot be ignored, but there is a lack of effective means of prevention and treatment. In our study, Shenyuan granules, by strengthening the body’s resistance and eliminating pathogenic factors, increased the level of serum sKl, inhibited the activation of Wnt/β-catenin, weakened the expression of Runx2, and improved vascular calcification. The research results provide potential targets and new ideas for the prevention and treatment of vascular calcification in CKD. However, immunohistochemical staining was not quantitatively detected in this study, and there is a lack of *in vitro* experiments to verify that sKl regulates Wnt/β-catenin. We look forward to further *in vitro* tests to confirm the effect of Shenyuan granules on the sKl/Wnt/β-catenin signaling pathway and the calcification of histocytes.

## Abbreviations

CKD: Chronic kidney disease
sKl: Soluble Klotho
CVD: Cardiovascular disease
VSMCs: Vascular smooth muscle cells
Wnt1: Wingless/Integrated 1
Runx2: Runt-related transcription factor 2
Sm22α: Smooth muscle 22 alpha
OCN: Osteocalcin
Ca: Calcium
BUN: Blood urea nitrogen
SCr: Serum creatinine
iPTH: Intact parathyroid hormone
ALP: Alkaline phosphatase
Sm22: Smooth muscle 22
